# Direct modulation of G protein-gated inwardly rectifying potassium (GIRK) channels

**DOI:** 10.3389/fphys.2024.1386645

**Published:** 2024-06-06

**Authors:** Ha Nguyen, Ian W. Glaaser, Paul A. Slesinger

**Affiliations:** Nash Family Department of Neuroscience, Icahn School of Medicine at Mount Sinai, New York, NY, United States

**Keywords:** G protein-gated inwardly rectifying potassium channel (GIRK), Kir3, PIP_2_, alcohol, cholesterol, small molecule modulator

## Abstract

Ion channels play a pivotal role in regulating cellular excitability and signal transduction processes. Among the various ion channels, G-protein-coupled inwardly rectifying potassium (GIRK) channels serve as key mediators of neurotransmission and cellular responses to extracellular signals. GIRK channels are members of the larger family of inwardly-rectifying potassium (Kir) channels. Typically, GIRK channels are activated via the direct binding of G-protein βγ subunits upon the activation of G-protein-coupled receptors (GPCRs). GIRK channel activation requires the presence of the lipid signaling molecule, phosphatidylinositol 4,5-bisphosphate (PIP_2_). GIRK channels are also modulated by endogenous proteins and other molecules, including RGS proteins, cholesterol, and SNX27 as well as exogenous compounds, such as alcohol. In the last decade or so, several groups have developed novel drugs and small molecules, such as ML297, GAT1508 and GiGA1, that activate GIRK channels in a G-protein independent manner. Here, we aim to provide a comprehensive overview focusing on the direct modulation of GIRK channels by G-proteins, PIP_2_, cholesterol, and novel modulatory compounds. These studies offer valuable insights into the underlying molecular mechanisms of channel function, and have potential implications for both basic research and therapeutic development.

## Introduction

**FIGURE 1:**
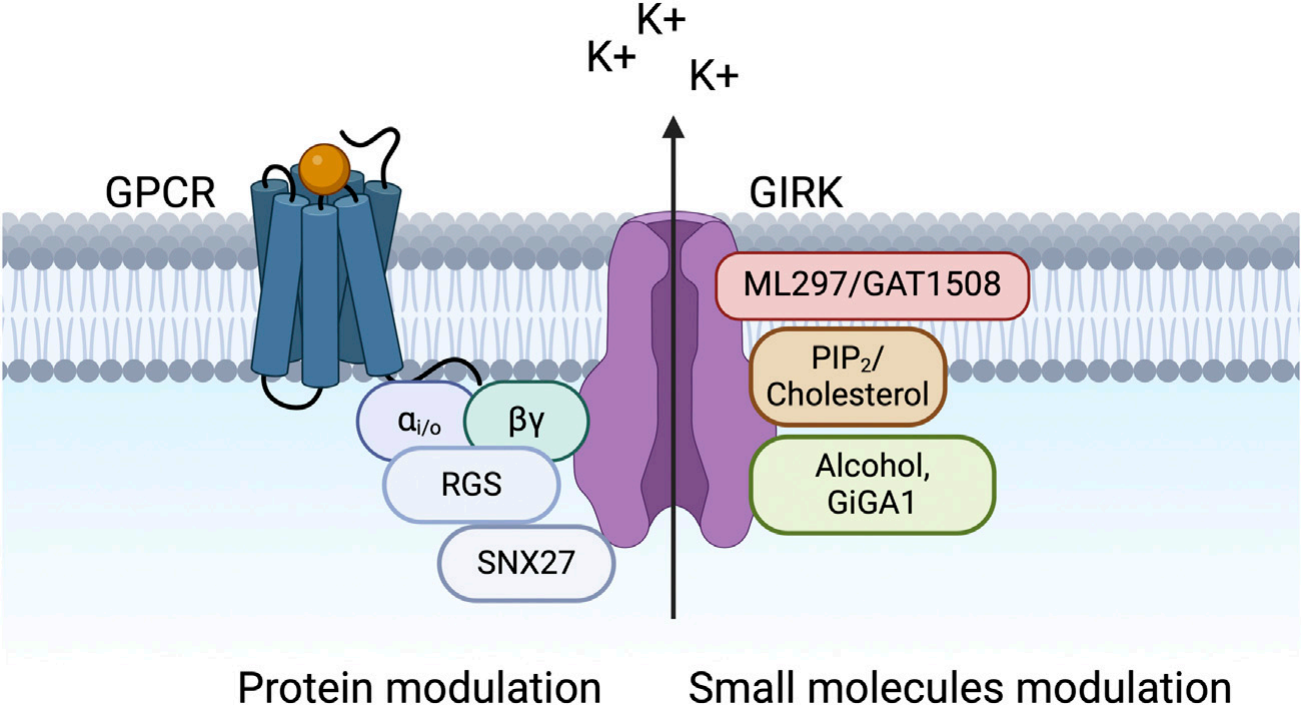
GIRK channels are modulated by various proteins and small molecules. The schematic shows a macromolecular signaling complex that contains a G protein-coupled receptor (GPCR), which couples to pertussis toxin (PTX)-sensitive G_i/o_ G proteins (Gα_i/o_ and Gβγ), a GIRK channel, a regulator of G protein signaling (RGS) protein, and sorting nexin 27 (SNX27). Small molecule modulators of GIRK channels are also shown, including PIP_2_, cholesterol, alcohol, and novel compounds (ML297, GAT1508, and GiGA1).

G protein-gated Inwardly Rectifying Potassium (GIRK) channels belong to an extensive family of inward rectifiers, denoted as Kir1 to Kir7. The term “inward rectification” signifies a sizeable inward current at a potential below the equilibrium potential for K^+^ (E_K_) (e.g., −85 mV) and a small outward current above E_K_. The outward current is reduced due to blockage of the channel’s pore by intracellular Mg^2+^ and polyamines at potentials above E_K_. GIRK channels are expressed in various tissues, including the nervous system, the heart, and the pancreas (Kurachi et al., 1986; Bond et al., 1995; Ferrer et al., 1995). Various neurotransmitters, including acetylcholine (ACh), dopamine, opioids, serotonin, somatostatin, adenosine, and γ-aminobutyric acid (GABA), stimulate their respective G protein-coupled receptors (GPCRs) (Luscher and Slesinger, 2010) which couple to pertussis toxin (PTX)-sensitive heterotrimeric G proteins (G_i/o_) (Figure 1). G protein Gβγ activation of GIRK channels then hyperpolarizes the membrane potential and reduces cell excitability.

**FIGURE 2:**
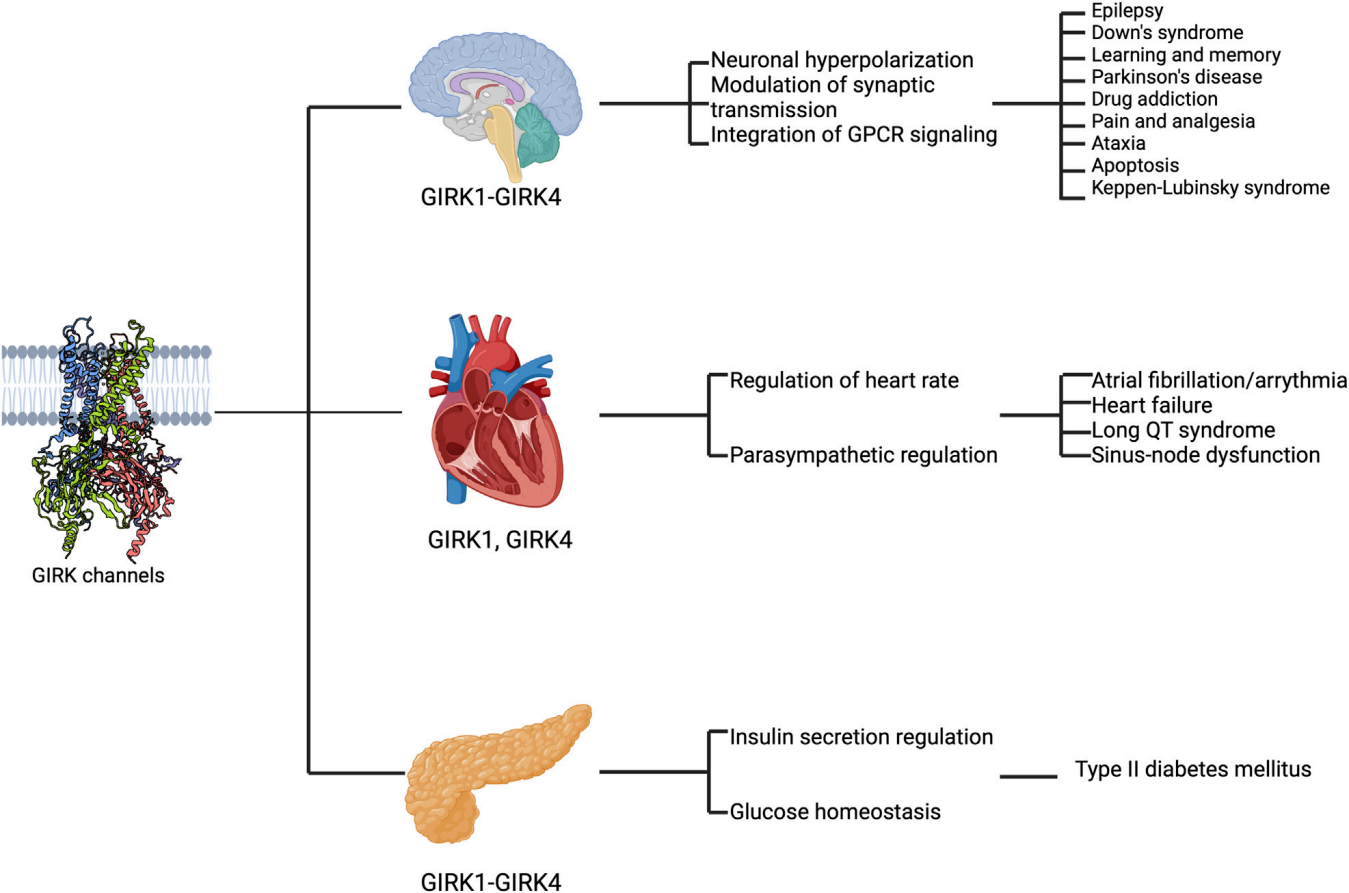
GIRK channels are expressed in various organs and implicated in disease states. The schematic highlights the expression of GIRK subunits in different regions (the brain, the heart, and the pancreas from top to bottom), their functional roles (first text column) and their implications in diseases (second text column).

Mammals express four subunits of GIRK channels: GIRK1 (Kir3.1/*KCNJ3*), GIRK2 (Kir3.2/*KCNJ6*), GIRK3 (Kir3.3/*KCNJ9*), and GIRK4 (Kir3.4/*KCNJ5*) (Luscher and Slesinger, 2010). GIRK channels function as tetramers in heterologous expression systems and native tissues (Liao et al., 1996; Jelacic et al., 2000; Tucker et al., 1996). GIRK1, GIRK2, and GIRK3 subunits are most prevalent in the brain (Karschin et al., 1996) (Figure 2). GIRK2 has three splice variants (GIRK2a-c), differing in the length of the C-terminal domain. Notably, GIRK2c contains a PDZ-binding motif absent in GIRK2a and GIRK2b (Lesage et al., 1994; Isomoto et al., 1996; Inanobe et al., 1999). GIRK2 possesses an endoplasmic reticulum (ER) export signal characterized by acidic residues and a valine-leucine (VL) dipeptide motif. These features allow the GIRK2 subunit to form either homotetramers or heterotetramers (Ma et al., 2002). Since GIRK1 and GIRK3 subunits lack an ER export signal, they cannot form functional homomeric channels on the plasma membrane. Instead, they combine to create heterotetrameric channels such as GIRK1/3, GIRK1/2, and GIRK2/3 (Kofuji et al., 1995; Ma et al., 2002). The main difference between GIRK1/2 and GIRK2 lies in their single-channel kinetics. GIRK1/2 heterotetramers exhibit markedly longer openings, evident by a 7-fold increase in the open-time duration compared to GIRK2 alone (Kofuji et al., 1995).

GIRK1 and GIRK4 are expressed in the heart and are often found together as GIRK1/4 heterotetramers (Krapivinsky et al., 1995; Mark and Herlitze, 2000), primarily composed of two GIRK1 and two GIRK4 subunits (Silverman et al., 1996; Corey and Clapham, 1998). Notably, GIRK4 also forms homotetramers (Corey and Clapham, 1998), found in atrial cells (Wickman et al., 1998) where they respond to acetylcholine released by the vagus nerve, leading to the activation of I_KACh_ (Dobrev et al., 2005). In cardiac pacemaker cells, intracellular Na^+^ regulates the sensitivity of homotetrameric GIRK4 channels to Gβγ subunits. In a reconstituted lipid bilayer system, binding of Na^+^ to GIRK4 enhances its affinity for Gβγ (Touhara et al., 2016). This observation is also true with GIRK2 in the brain (Wang et al., 2016). In contrast, GIRK1 lacks a functional Na^+^ binding site (Ho and Murrell-Lagnado, 1999) but it might regulate the affinity of GIRK1/4 to Gβγ by mimicking the Na^+^ -bound GIRK4 subunit (Touhara et al., 2016).

The relative abundance between GIRK1/4 and GIRK4 in cardiac cells remains unclear (Touhara et al., 2016). Additionally, there is ambiguity regarding the extent of functional differences between these channels due to the challenge of isolating GIRK1/4 channels for study, as heterologous expression of both subunits inherently results in a mixed population of homo- and heterotetramers (Touhara et al., 2016). The response of GIRK4 channels can be modulated by intracellular Na^+^ concentration, while GIRK1/4 channels remain unaffected by intracellular Na^+^ levels, signaling a state of rest (Touhara et al., 2016).

The function and trafficking of GIRK channels are closely tied to their subunit composition. For example, GIRK2 is responsible for most of the GIRK currents in the brain (Luscher et al., 1997; Torrecilla et al., 2002; Koyrakh et al., 2005), with the GIRK1/2 heterotetramer being the most abundant (Liao et al., 1996). GIRK3 contains a lysosomal targeting motif, reducing GIRK channel expression in the plasma membrane by redirecting GIRK heterotetramers towards lysosomes for degradation (Ma et al., 2002). In addition, GIRK3 regulates the GIRK signals in the brain by associating with the trafficking protein sorting nexin 27 through a PDZ-binding motif in its C-terminal tail (Lunn et al., 2007; Balana et al., 2011; Munoz and Slesinger, 2014).

GIRK channels exhibit considerable structural similarity to other Kir channels. Each subunit consists of two integral membrane-spanning segments, a reentrant P-loop that forms the selectivity filter, and cytoplasmic amino- and carboxy-termini (Ho et al., 1993; Kubo et al., 1993; Doupnik et al., 1995). GIRK channels possess two operational gates–an inner helix gate (also known as helix bundle crossing gate, formed by four F192 side chains in the inner helices of GIRK2) and a G-loop gate (formed by hydrophobic residues on the βH-βI loop of the CTD) (Pegan et al., 2005)–both of which are controlled by a combination of stimuli originating from the cytoplasmic and membrane environments (Whorton and MacKinnon, 2011).

The selectivity filter of GIRK channels contains a distinctive amino acid sequence (TVGYG) that dictates the selectivity of most K^+^ channels. This motif forms a pore that favors the movement of unhydrated K^+^ (Roux and MacKinnon, 1999; Morais-Cabral et al., 2001). Larger ions such as Cs^+^ and TEA block the channel from the inner side. In contrast, unhydrated ions smaller than K^+^, such as Na^+^, cannot effectively coordinate in the pore without high energy costs. Based on permeation studies, the diameter of the selectivity filter was estimated to be 2.6–3 Å, and the inner pore of the channel was projected to have a diameter of 8 Å (Bezanilla and Armstrong, 1972).

In the nervous system, GIRK channels regulate neuronal excitability through GIRK-mediated self-inhibition (Lacey et al., 1987; Bacci et al., 2004), slow inhibitory synaptic potentials (Isaacson et al., 1993; Scanziani, 2000; Nicoll, 2004; Huang et al., 2005; Fernandez-Alacid et al., 2009), and volume transmission (Sun et al., 2002; Bacci et al., 2004). Pharmacological and animal studies on GIRK channels suggest that GIRK activity is crucial in pain perception (Ikeda et al., 2000; Blednov et al., 2003; Mitrovic et al., 2003) as well as learning and addiction (Tipps and Buck, 2015). Additionally, abnormal GIRK activity has been implicated in human diseases such as epilepsy (Signorini et al., 1997; Pei et al., 1999; Mazarati et al., 2006), Down Syndrome (Reeves et al., 1995; Sago et al., 1998; Siarey et al., 1999), Parkinson’s disease (Harkins and Aaron, 2002; Coulson et al., 2008), and drug addiction (Hill et al., 2003; Morgan et al., 2003; Kozell et al., 2009). Mutations at conserved amino acids in GIRK channels can result in a loss of K^+^ selectivity, disrupting cell function (Patil et al., 1995; Kofuji et al., 1996) and causing cell death (Navarro et al., 1996; Roffler Tarlov et al., 1996; Slesinger et al., 1996; Choi et al., 2011; Scholl et al., 2012; Horvath et al., 2018). For instance, *de novo* mutations in the human GIRK2 channel (encoded by *KCNJ6*), such as G154S (p.Gly154Ser), T152Δ (three-nucleotide deletion causes in-frame heterozygosity, resulting in the loss of one amino acid, p. Thr152del) (Masotti et al., 2015), and L171R (p.Leu171Arg) (Horvath et al., 2018), lead to Keppen-Lubinsky syndrome (KPLBS) or a KPLBS-like disorder (Figure 2).

The autonomic control of heart rate includes both sympathetic and parasympathetic regulation of cardiac ion channels through separate GPCR signaling pathways. In the parasympathetic modulation of heart rate, acetylcholine plays a crucial role in activating muscarinic m2 receptors, which are coupled to GIRK channels expressed in pacemaker cells of the sinoatrial and atrioventricular nodes, as well as atrial cardiomyocytes (Pfaffinger et al., 1985; Logothetis et al., 1987). The activation of cardiac GIRK channels by acetylcholine leads to a slowing of pacemaker action potential firing by increasing the potassium conductance across the cell membrane and hyperpolarizing the cell membrane potential (Dascal, 1997; Kovoor et al., 2001; Mesirca et al., 2014). Besides GIRK activity caused by neural stimulation, cardiac GIRK channels also exhibit constitutive activity, which can be attributed to the different GIRK subunits (Voigt et al., 2014). Elevated constitutive GIRK signals are shown to have proarrhythmic effects in patients with atrial fibrillation (Dobrev et al., 2005; Voigt et al., 2008) and in the heart failure mouse model (An and Cho, 2023) (Figure 2).

In the pancreas, GIRK channels contribute significantly to regulating insulin secretion. GIRK channels are downstream effectors of hormones such as noradrenaline (Bünemann et al., 2001), somatostatin (Kreienkamp et al., 1997; Yoshimoto et al., 1999; Smith et al., 2001; Kailey et al., 2012), galanin (Smith et al., 1998), and adrenaline (Iwarnir and Reuveny, 2008). These agents influence the pancreatic β-cells through various mechanisms (Sharp, 1996), including one in which increased K^+^ efflux induced membrane hyperpolarization (Rorsman et al., 1991; Smith et al., 2001; Sieg et al., 2004). There is evidence that all four subunits of GIRK channels are expressed at varying levels in the pancreas (Tanizawa et al., 1996), pancreatic islet cells (Ferrer et al., 1995; Yoshimoto et al., 1999), and insulinoma (Bond et al., 1995). Dysfunctional GIRK channels in the pancreas have been implicated in disorders such as diabetes (Stoffel et al., 1995; Vaughn et al., 2000), highlighting their importance in glucose homeostasis (Figure 2). In the adrenal gland, mutations such as G151R, T158A, or L168R in the human GIRK4 channel (encoded by *KCNJ5*) result in aldosterone-producing adenomas (APA) (Boulkroun et al., 1979; Choi et al., 2011).

Numerous modulators that influence the activity of GIRK channels have been identified, including sodium, ethanol, and phosphorylation by protein kinase A (PKA) and protein kinase C (PKC). Both Na^+^ and ethanol enhance GIRK signals through specific binding sites on the channel (Aryal et al., 2009; Whorton and MacKinnon, 2011). Phosphorylation by PKC reduces channel activity, while PKA-dependent phosphorylation increases channel activity. Moreover, alterations in the levels of the membrane phospholipid phosphatidylinositol 4,5 bisphosphate (PIP_2_) can regulate GIRK channel activity (Huang et al., 1998; Wang et al., 2014; Glaaser and Slesinger, 2017). Stimulation of GPCRs linked to G_q_ proteins activate phospholipase C (PLC), depleting plasma membrane PIP_2_ levels and reducing GIRK currents. PLC activation also stimulates PKC, establishing cross-talk between PIP_2_ depletion and PKC phosphorylation in regulating GIRK channels. Finally, due to emergence of GIRK channels as potential drug targets, several novel compounds (including ML297, GAT1508, and GiGA1) that regulate GIRK channel activity have been identified. These compounds have shown promise in vivo studies examining antiseizure, anxiolytic, and fear extinction effects (Table 1) (Kaufman et al., 2013; Xu et al., 2020; Zhao et al., 2020).

**TABLE 1:** Pharmacokinetics and biological activity of small molecule GIRK modulators observed in heterologous expression systems and in native GIRK-Expressing neurons.

Compound	GIRK selectivity	EC_50_	pK (brain to plasma ratio)	GIRK activity in HEK293 cells	GIRK activity in animals	Behavioral effect in animals
**ML297** Kaufmann et al. (2013); Wydeven et al. (2014)	GIRK1-containing channels (preference for GIRK1/2	110 ± 13 nM	0.2	↑↑↑ GIRK1/2 ↑ ↑ GIRK1/4	↑ GIRK currents similar to baclofen-induced currents in mouse hippocampal neurons ↓ neuronal excitability in mouse hippocampal neurons ↓ neuronal excitability in mouse hypothalamic and hippocampal brain slices	• Antiepileptic effect • Anxiolytic effect • Stress-induced hyperthermia reduction
**GAT1508** Xu et al. (2020)	GIRK1/2	75 ± 10 nM	ND	↑ GIRK1/2 NC GIRK1/4	↑ outward current in rat BLA brain slices	• Fear extinction effect
**GiGA1** Zhao et al. (2020)	GIRK1-containing channels (preference for GIRK1/2)	31 uM	1.91 ± 0.86	↑↑↑ GIRK1/2 ↑ GIRK1/3 and GIRK1/4	↑ GIRK currents similar to baclofen-induced currents in mouse hippocampal brain slices ↓ neuronal excitability in mouse hippocampal brain slices	• Antiepileptic effect

## Lipid modulation of GIRK channels

### PIP_2_ is necessary for GIRK channel activation

PIP_2_ is a crucial phosphoinositide (PIP) in the plasma membrane. When specific cell surface receptors are activated, phospholipase C (PLC) activation stimulates the hydrolysis of PIP_2_ into IP_3_ and DAG. IP_3_ triggers the release of calcium ions from intracellular stores, while DAG activates protein kinase C (PKC), leading to a cascade of intracellular signaling events.

In 1998, findings from separate laboratories indicated that the activation of G protein-sensitive channels relied on the presence of PIP_2_ (Huang et al., 1988; Sui et al., 1998). A PIP_2_-specific antibody or activation of PLC impeded channel activity. Conversely, GIRK channel activity can only be restored by Gβγ or Na^+^ in the presence of PIP_2_ (Sui et al., 1988; Huang et al., 1998). Notably, PIP_2_ alone can activate GIRK1/4 channels within minutes, which is accelerated by adding Gβγ (Huang et al., 1998). These results suggested that Gβγ stabilizes the interactions between PIP_2_ and the GIRK channel. Although, the necessity for PIP_2_ was apparent, the mechanism through which PIP_2_ affected channel activation was, at that time, unclear.

In fact, the interaction with PIP_2_ is required for the activation of all inward rectifiers. Nevertheless, different channels in the same family exhibit varying affinities for PIP_2_. GIRK channels exhibit lower specificity and weaker affinity to PIPs compared to most other Kir channels (Rohacs et al., 2003). This characteristic can explain the low open probability of GIRK channels in single-channel recordings (Kofuji et al., 1995) and the involvement of other intracellular activators like Gβγ, Na^+^, and ethanol for robust channel activity.

In 2011, Whorton and MacKinnon reported the atomic structure of the GIRK2 channel in association with PIP_2_. PIP_2_ binds to the channel at the interface between the transmembrane domain (TMD) and the cytoplasmic domain (CTD). The negatively charged phosphates of the PIP_2_ molecule are coordinated by positively charged residues, including K64, K194, K199, and K200 at the interfacial helices. Residues K90 and R92 at the outer helices also form electrostatic interactions with the ligand. Notably, the crystal structure revealed that the G-loop gate and inner helix gate are too constricted to conduct hydrated K^+^ ions, suggesting the channel in the presence of PIP_2_ is closed in this structure (Whorton and MacKinnon, 2011).

However, there is evidence indicating that PIP_2_ is sufficient for channel activation. Two groups reconstituted purified GIRK2 channels into liposomes containing brain PIP_2_ and observed potassium conductance in the presence of PIP_2_ alone (Whorton and MacKinnon, 2011; Glaaser and Slesinger, 2017). This effect was inhibited by adding a GIRK2 inhibitor (MTS-HE) or the K^+^ channel inhibitor BaCl_2_. Additionally, the acute addition of diC8-PIP_2_ directly activated GIRK2 channels in a dose-dependent manner. These findings indicate that both brain PIP_2_ and soluble diC8-PIP_2_ can activate GIRK2 channels, suggesting direct gating by PIP_2_ in the presence of Na^+^ without the need for G proteins or other ligands (Glaaser and Slesinger, 2017). Although these experiments indicate that PIP_2_ acts as an agonist and is sufficient for channel activation in liposomes, other studies assaying GIRK channels in lipid bilayers suggest that PIP_2_ is not sufficient for channel opening (Wang et al., 2014; 2016). Finally, GIRK channels expressed in HEK293 cells demonstrate basal K^+^ currents that can be reduced by the application of a voltage-activated phosphatase (Dr-VSP), implying that there is a PIP_2_-mediated basal current in cells (Bodhinathan and Slesinger, 2013).

Mutagenesis studies on residues interacting with PIP_2_ provide molecular insights into PIP_2_-dependent activation of GIRK channels. Substituting K200 in GIRK2 with an uncharged residue (i.e., Tyr) causes an increase in channel activity, possibly due to the change in the PIP_2_ affinity of the mutated GIRK channel (Lacin et al., 2017). Molecular dynamic (MD) simulations indicate a more vital PIP_2_ interaction of the mutant channel (K200Y). These results suggest that the highly conserved positively charged residue K200 in GIRK2 supports a dynamic interaction with PIP_2_ that decreases the channel’s open probability in the absence of other modulators (Gβγ, ethanol) (Lacin et al., 2017). Another group showed that specific point mutations in the PIP_2_ binding site alter the selectivity of GIRK2 for different PIPs (Qiao et al., 2020). For example, the K64Q mutation displays the highest affinity for PI(4,5)P_2_ with a preference for particular acyl chains, the R92P mutation results in the relatively non-specific binding of various PIP isoforms, and the K194A mutation exhibits a binding preference for PI(3,4,5)P_3_ over other PIPs (Qiao et al., 2020).

**FIGURE 3:**
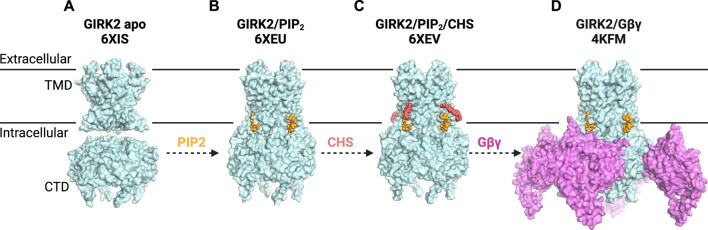
G Protein-Gated Inwardly Rectifying Potassium (GIRK) Channel Structures and Modulatory Sites. **(A)** structure of GIRK2 apo (PDB: 6XIS) shows the CTD disengaged from the TMD (disordered linkers not shown). **(B)** structure of GIRK2 in the presence of PIP_2_ (yellow) (PDB: 6XEU) suggests a portion of GIRK2/PIP_2_ particles adopt an engaged or docked CTD. **(C)** structure of GIRK2 in complex with PIP_2_ (yellow) and CHS (salmon) (PDB: 6XEV) demonstrates that PIP_2_ binds at its canonical site, while CHS can be docked near the PIP_2_ binding site. CHS potentially stabilizes the channel-PIP_2_ interactions and increases the portion of engaged-CTD particles. **(D)** structure of GIRK2 with four Gβγ subunits (lilac), four PIP_2_ molecules (yellow) and Na^+^ ions (not visible) (PDB: 4KFM) reveals the interplay of these modulators in GIRK2 gating.

Recent structural studies have shed new light on the role of PIP_2_ in channel activation. In contrast to the X-ray structure of GIRK2 apo (Whorton and MacKinnon, 2011), where the CTD is engaged with the TMD in the apo state, cryo-EM structures showed the CTD distancing from the TMD in the apo state (the extended TM-CTD) (Figure 3). The observed difference could be attributed to the lattice packing in X-ray crystal structures. The single-particle analysis demonstrates that PIP_2_ alters the equilibrium between two distinct structures of neuronal GIRK2: the extended and docked TM-CTD, favoring the docked conformation with increasing concentrations of PIP_2_. PIP_2_ binding leads to a reconfiguration of the Gβγ binding surface on the cytoplasmic domain (movement of the βL-βM loop), priming it to interact with Gβγ (Niu et al., 2020).

In the same effort to investigate the structure of GIRK channels in the absence and presence of PIP_2_, Mathiharan et al. also observed that the CTD is separated from the TMD in both the GIRK2 apo state and a substantial portion of GIRK2/PIP_2_ particles (Mathiharan et al., 2021) (Figure 3). Despite the apparent detachment of the CTD, it is connected by a stretched tethered linker that remains unresolved. The presence of PIP_2_ appeared to increase the percentage of channels in the docked state. Structures of GIRK2 with PIP_2_ and the cholesterol analog cholesteryl hemisuccinate (CHS) demonstrated even further stabilization of the docked state. The pore diameter at the inner helix (F192) and G-loop (M313) gates is approximately 6.5 Å and 4 Å, respectively, which is insufficient for hydrated K^+^ (8 Å) to permeate (Mathiharan et al., 2021). However, previous research using crosslinked KirBac3.1 channels (Black et al., 2020) and MD simulations of GIRK channels (Bernsteiner et al., 2019) suggests that this degree of opening at the inner-helix gate may permit the passage of partially hydrated or transiently non-hydrated K^+^.

Interestingly, though the structures of GIRK channels look similar, the proposed GIRK channel gating mechanism models are different. MacKinnon and colleagues propose that although four PIP_2_ molecules bind to GIRK2, the pore remains closed due to a constricted G-loop gate (Niu et al., 2020). On the other hand, Slesinger and colleagues suggest that PIP_2_ binding triggers a significant conformational shift in GIRK2, characterized by a “twisting engagement” followed by a “locking rotation” of the CTD with the TMD after engagement. This rotational locking is crucial for opening the G-loop and helix bundle crossing (HBC) gates (Mathiharan et al., 2021). Notably, this observation potentially clarifies why GIRK channels do not open without PIP_2_, even when exposed to Gβγ, ethanol, or cholesterol.

Additionally, several groups have used MD simulations to provide a nuanced understanding of the gating mechanism of GIRK channels. For instance, Li et al. (2020) explored the permeation process through the GIRK2 selectivity filter (SF) and the open HBC gate. Their findings indicated that permeation efficiency upon channel activation is predominantly determined by the SF rather than the HBC or the G-loop gate. A study by Li et al. (2019) revealed a synergistic effect of Na^+^ and Gβγ to control the opening of the HBC gate: the binding of Na^+^ to the CD loop triggers interactions leading to G-loop gate stabilization, while Gβγ induces a rocking motion of the CTD, which stabilizes GIRK2 interactions with PIP_2_.

In the last decade, structural insights into GIRK channels and their interactions with a diverse group of intracellular regulators (PIP_2_, Na^+^, Gβγ) GIRK2 channels have advanced significantly. However, a comprehensive structural model of all GIRK channels and modulators is still lacking. There is an overrepresentation of GIRK2 homotetramers in cryo-EM studies. The success of expression and purification of cardiac GIRK1/4 heterotetramers (Touhara et al., 2016; Niu et al., 2020) is promising and may lead to new structural information on GIRK channels. However, it is also worth noting that structures of GIRK channels purified in detergent might be different from those in their native lipid environment. In the future, reconstituting GIRK channels in nanodiscs could provide novel mechanistic insights into GIRK channel gating.

### Cholesterol potentiates GIRK currents in a G protein-independent manner

Cholesterol is a significant constituent of the cell membrane and plays a crucial role in numerous cellular functions and cell growth. Cholesterol exhibits uneven distribution between the plasma and intracellular membranes, with most free cholesterol (40%–90%) located in the plasma membrane (Slotte and Bierman, 1988; Lange, 1991). All cholesterol in the CNS is synthesized *de novo* (Jeske and Dietschy, 1980; Zhang and Liu, 2015; Jin et al., 2019), whereas the cholesterol level in the periphery depends on diet and production in organs (Grundy, 1983; Dietschy et al., 1993; Dietschy, 1997). Excessive cholesterol levels are linked to various pathological conditions (Yeagle, 1991; Kellner-Weibel et al., 1999), including the onset of cardiovascular disease (Steinberg, 2002) as well as neurodegenerative diseases like Alzheimer’s (Cho et al., 2019) and Parkinson’s disease (Paul et al., 2017).

Changes in cholesterol levels influence the operation of an expanding array of ion channels (Bowles et al., 1985; Wu et al., 1995; Levitan et al., 2000; Romanenko et al., 2004; Rosenhouse-Dantsker et al., 2012). In functional assays, cholesterol can activate some ion channels (Lockwich et al., 2000; Shlyonsky et al., 2003) and inhibit others (Bolotina et al., 1989; Romanenko et al., 2004; Toselli et al., 2005). For example, cholesterol suppresses the activity of Kir1.1, Kir2.1, Kir4.1, GIRK1 (with the pore mutation F137S), and Kir6.2Δ36 (the C-terminal truncation mutant of Kir6.2) (Rosenhouse-Dantsker et al., 2010). On the other hand, an increase in cholesterol level results in the activation of GIRK2 channels in the hippocampus and GIRK4 channels in the atrial myocytes (Rosenhouse-Dantsker et al., 2010; Bukiya and Rosenhouse-Dantsker, 2017; Rosenhouse-Dantsker, 2019; Corradi et al., 2022). With purified GIRK2 channels reconstituted in liposomes, it was shown that cholesterol directly potentiates GIRK channel activity in a PIP_2_-dependent manner (Glaaser and Slesinger, 2017). In planar lipid bilayers, PIP_2_ and cholesterol synergistically activate GIRK4 (Bukiya and Rosenhouse-Dantsker, 2017). Some GIRK channels are found in lipid rafts with high concentrations of cholesterol (Delling et al., 2002) and can reciprocally influence the cholesterol distribution in the membrane (Corradi et al., 2022).

The presumed cholesterol binding sites in GIRK channels were believed to be in the same regions as those in Kir2.1, situated between the inner and outer transmembrane helices (Rosenhouse-Dantsker et al., 2010; Rosenhouse-Dantsker et al., 2013; Rosenhouse-Dantsker, 2018). However, specific residues constituting the binding pockets in each channel were found to be different. For instance, mutations that did not affect Kir2.1’s response to cholesterol significantly impacted the cholesterol sensitivity of the GIRK2 channel. Additionally, mutations in the outer transmembrane helix of GIRK2 did not exhibit the same cholesterol effects as the equivalent mutations in GIRK4 channels, indicating differences in their respective binding pockets (Rosenhouse-Dantsker et al., 2010). GIRK2 was suggested to possess two potential cholesterol-binding sites: a primary one at the center of the transmembrane domain (V99 and L174) and a second one near the TMD-CTD interface (L86, V101, and V183) (Bukiya et al., 2017; Rosenhouse-Dantsker, 2019). Due to the absence of a known structure, further investigations are required to identify the putative cholesterol-binding sites in other GIRK subunits.

To examine the structural role of cholesterol, Mathiharan et al. determined the cryo-EM structure of GIRK2 with PIP_2_ and cholesteryl hemisuccinate (CHS), an analog of cholesterol (Figure 3). The structure revealed that PIP_2_ binds at its canonical site near the inter-domain linkers. CHS molecule can be docked in two planar densities on the opposite side of the PIP_2_-channel interface. The first density has the head group of CHS forming a salt bridge with R92. At the same time, its sterane rings and isooctyl tail were stacked against the TMD, surrounded by hydrophobic residues from adjacent subunits. Mutations of key residues (F93, L95, L65, V99), predicted from the structure to mediate channel-cholesterol interactions, ablated cholesterol potentiation in a heterologous expression system (Mathiharan et al., 2021). These results provided functional evidence with cholesterol confirming the GIRK2-CHS interactions observed in the structure. Notably, the locations of CHS in the cryoEM GIRK2 structures differed from those identified through computational molecular docking (Bukiya et al., 2017). It is plausible that cholesterol binds to various regions in the channel in different lipid membrane environments. Combined with the fact that cholesterol potentiates the GIRK2 channel in the presence of PIP_2_, it can be inferred that the prominent role of CHS/cholesterol is to augment PIP_2_ binding (Glaaser and Slesinger, 2017; Mathiharan et al., 2021).

Elevated plasma cholesterol linked to cardiac diseases is well-established (Rosenson et al., 2018). However, the role of cholesterol in the nervous system remains poorly understood. With its implication in some neurodegenerative diseases (Vance, 2012; Paul et al., 2017; Cho et al., 2019), it is important to determine the impact of cholesterol on brain functionality, including GIRK channels.

## Protein modulation of GIRK channels

### Gβγ activates GIRK channels

Most G protein-signaling pathways involve the exchange of GTP for GDP upon the binding of a receptor to a partner G protein. This exchange triggers the dissociation of the trimeric G protein into α and βγ subunits. Subsequently, the Gα subunit activates or inhibits second messengers like adenylyl cyclase or phospholipase C, which will then act on downstream effectors. GIRK channels represent one of the first direct effectors of G proteins. GIRK channels are activated via a membrane-delimited pathway, restricted to a two-dimensional membrane microdomain, where GPCRs and GIRK channels are either nearby or form a stable signaling complex without the need for soluble cytosolic secondary messengers (Pfaffinger et al., 1985; Soejima and Noma, 1984). The GPCR-GIRK cascade is a primary transducer of inhibitory effects from neurotransmitters such as ACh, dopamine, GABA (through GABA_B_ receptors), opioids, serotonin, adenosine, and others.

To investigate whether G proteins directly activated GIRK channels, one group demonstrated that Gα pre-activated with GTPγS (Gα-GTPγS) robustly activated GIRK channels (Codina et al., 1987). Another group found that the application of purified Gβγ subunits, not Gα-GTPγS, directly activated GIRK channels (Logothetis et al., 1987). With the advancement in techniques and the cloning of GIRK subunits, there is a consensus that Gβγ subunits activate GIRK channels (Reuveny et al., 1994; Wickman et al., 1994; Whorton and Mackinnon, 2013). Conversely, mutations in either Gβ or Gγ subunit negatively impact the ability of G proteins to regulate GIRK signals (Peng et al., 2003; Zhao et al., 2003).

Although no further experiments have demonstrated Gα activation of GIRK channels, Gαi_/o_ proteins are still recognized as important regulators, influencing receptor specificity and basal channel activity (Peleg et al., 2002; Rishal et al., 2005; Rubinstein et al., 2007). Researchers characterized the low binding affinity of Gβγ subunit to GIRK channels as well as a much higher binding affinity of Gβγ to the Gα subunit, which leads to a possibility that Gα is tethered to Gβγ when interacting with the GPCR-GIRK channel macromolecular complex (Clancy et al., 2005; Berlin et al., 2010; Wang, Whorton and MacKinnon, 2014).

In the effort to characterize the binding site of Gβγ in GIRK channels, cross-linking experiments revealed the binding of four Gβγ subunits per tetramer, with each Gβγ binding to one channel subunit (Corey and Clapham, 2001; Sadja et al., 2002). The putative Gβγ binding sites are localized to specific residues on the N- and C-termini of the GIRK1/2 subunits (Huang et al., 1995; Huang et al., 1997; Ivanina et al., 2003; Finley et al., 2004). Mutagenesis studies indicated that L344E in GIRK2 was particularly important in Gβγ activation (Finley et al., 2004). Mutagenesis studies demonstrated that H64 in the N-terminus and L268 in the C-terminus of the GIRK4 subunit significantly contributed to the Gβγ sensitivity in GIRK1/4 heterotetramers (He et al., 2002). Mutations at the equivalent residues in the GIRK1 subunit also appeared to be involved in the Gβγ-mediated channel activity (He et al., 2002). A different approach looking at mutations in G protein also pinpointed regions of Gβγ that interact with GIRK channels (Zhao et al., 2003).

Two primary models have been proposed to explain the gating mechanism of GIRK by Gβγ. Reuveny and colleagues utilized a tandem tetrameric approach, revealing a graded contribution of one to four Gβγ subunits binding to allow partial to full channel openings (Sadja et al., 2002). This model was further corroborated by the Dascal group, demonstrating that increased Gβγ expression is constitutively linked with elevated GIRK channel expression levels (Rishal et al., 2005). Alternatively, the second model posits that all four Gβγ subunits are necessary for channel activation. MacKinnon and colleagues discovered that four Gβγ subunits cooperatively bind to open GIRK2 channels in the presence of PIP_2_, with intracellular Na^+^ enhancing channel opening primarily by increasing Gβγ affinity (Wang et al., 2014; 2016). Their crystallographic analysis of the mammalian GIRK2 channel at a resolution of 3.5 Å also demonstrated the stabilization of four Gβγ subunits at the interfaces between the four K^+^ channel subunits (Figure 3). Specifically, Q248 on the βD-βE loop and residues 342–344 on the βL-βM loop of GIRK2 form bonds with different regions of Gβ (Whorton and Mackinnon, 2013). In addition to the direct engagement, there is a possibility that long-range electrostatic interactions between G protein and GIRK2 occur when Gβγ is released upon GPCR stimulation. The binding of Gβγ causes a conformational change in GIRK2 that relaxes the HBC gate (formed by four F192 residues on the inner helices), widening it to 6–7 Å (Whorton and Mackinnon, 2013). Compared to structures of fully open K^+^ channels, the crystal structure of GIRK2/Gβγ is considered a pre-open state of the channel. This pre-open state displays a conformation intermediate between the closed and the constitutively active mutant R201A open conformations. The resulting structural depiction aligns with the “membrane delimited” activation of GIRK channels by G proteins and the characteristic burst kinetics observed in channel gating (Whorton and Mackinnon, 2013).

While crystallography and cryo-EM studies have significantly advanced the understanding of GIRK-Gβγ interactions, the truncation of the NTD and CTD remains a gap in comprehending GIRK channel gating mechanisms. Notably, several investigations underscore the significance of the distal CTD of GIRK1 in channel function. Ivanina et al. revealed that although both GIRK1 and GIRK2 subunits bind Gβγ at the N-terminus, GIRK1 possesses an additional Gβγ-interacting segment in its first half of the C-terminus. Moreover, mutations in specific C-terminal leucine residues of GIRK1 altered channel properties without affecting Gβγ binding, indicating their role in modulating Gβγ-induced changes in channel gating (Ivanina et al., 2003). Furthermore, residue Q404 within GIRK1’s CTD was identified as influencing receptor-activated channel activity (Wydeven et al., 2012).

GIRK channels exhibit a low open channel probability (Kofuji et al., 1995). This characteristic may explain why existing structures of GIRK, which predominantly captured channel particles in a closed state, cannot portray a fully open channel. Additionally, proteins contributing to the GIRK activation complex, such as RGS and Gα, could offer valuable structural insights. Structural studies of these proteins with GIRK channels might provide additional comprehensive evidence on G protein-mediated channel gating mechanism. To this point, the structures of GIRK2 imply that when PIP_2_ binds at the interface of the transmembrane and cytoplasmic domains, it engages both domains, which are normally separated in closed channels, thereby priming the channel for opening. PIP_2_ also confers conformational changes on βL-βM loop that are favorable for Gβγ binding, ultimately resulting in the expansion of the channel pore (Figure 3).

### Modulatory effects of regulators of G protein signaling (RGS) proteins on GIRK channels

Regulators of G protein signaling (RGSs) constitute a large family of proteins that are involved in GPCR-GIRK signaling (Ross and Wilkie, 2000; Hollinger and Hepler, 2002). RGS proteins contain a helical bundle made of approximately 110 conserved amino acids, known as the RGS domain (Nance et al., 1993; Berman et al., 1996). The RGS domain can bind to the Gα subunit and significantly enhance GTP hydrolysis (Riddle et al., 2005; Arshavsky and Wensel, 2013; Stewart and Fisher, 2015). The human genome encompasses over 30 genes encoding RGS or RGS-like domains, organized into at least six distinct subfamilies based on amino acid sequence similarity (Hollinger and Hepler, 2002; Willars, 2006). Several RGS transcripts undergo extensive alternative splicing, contributing to a diverse array of RGS protein expressions (Gold et al., 1997; Larminie et al., 2004; Xie and Palmer, 2007). Multiple RGS proteins are expressed in the brain and the heart (Dohlman et al., 1996; Zhang and Mende, 2011; Perschbacher et al., 2018).

The RGS proteins exert negative and positive regulation depending on the effectors involved. For instance, RGS proteins negatively modulate μ-opioid receptor inhibition of evoked GABA release (Clark et al., 2003; 2004; Zachariou et al., 2003; Lamberts et al., 2013) in the ventrolateral periaqueductal gray (vlPAG). Conversely, RGS proteins serve as activators for certain effectors of the μ-opioid receptor. As μ-opioid receptors induce neuronal hyperpolarization through the activation of GIRK channels in the vlPAG (Chieng and Christie, 1996; Ingram et al., 2008), RGS proteins positively regulate the coupling between μ-opioid receptors and GIRK channels in the vlPAG (McPherson et al., 2018).

Expression of most RGS proteins from the R4 and R7 families in heterologous systems induces various alterations in GIRK channel function (Doupnik et al., 1997; Chuang et al., 1998; Zhang et al., 2002). RGS facilitates the sequestration of free Gβγ dimers by accelerating the GTP hydrolysis of Gα subunits, leading to a notable reduction in the receptor-independent basal GIRK channel activity (Doupnik et al., 1997; Zhang et al., 2002). The R7 proteins (including RGS6, RGS7, RGS9, and RGS11) exhibit selectivity for Gα_o_ and engage unique binding partners, such as the Gβ5 subunit (Slepak, 2009). Gβ5 binds to a GGL domain in all four R7 RGS isoforms, stabilizing the native protein complex (Snow et al., 1998). The R4 subfamily (comprising RGS1-5, 8, 13, 16, 18, and 21) consists of the smallest-sized RGS proteins (Bansal et al., 2007) and demonstrates specificity towards G_i_ (Asli et al., 2021). Despite their compact size, these proteins have been implicated in pathological diseases such as type II diabetes (Zhang et al., 2022) and heart failure (Borges et al., 2023).

RGS7, a protein in the R7 subfamily, is involved in the signaling of GABA_B_ receptor-GIRK channels (Xie et al., 2010; Ostrovskaya et al., 2018). Subsequent investigations in RGS7^−/−^ mice reveal that the absence of RGS7 leads to a deceleration in the deactivation kinetics of GABA-activated GIRK channels in hippocampal neurons (Ostrovskaya et al., 2014). A significant factor influencing the modulation of neuronal R7 is the R7-binding protein (R7BP). R7BP engages in protein-protein interactions with the DEP (Dishevelled, Egl-10, and Pleckstrin domain) present at the N-terminus of all R7 proteins (Drenan et al., 2005; Hepler, 2005; Jayaraman et al., 2009; Liapis et al., 2012). In addition to facilitating membrane localization, R7BP safeguards R7-Gβ5 complexes from proteolytic degradation, consequently enhancing the steady-state cellular concentrations of R7-Gβ5 (Anderson et al., 2007). Expressing R7BP in a heterologous system has been demonstrated to significantly boost the acceleration of GPCR-GIRK channel gating kinetics by RGS7-Gβ5 (Drenan et al., 2005; 2006; Ostrovskaya et al., 2018).

Another R7 RGS protein, RGS6, appears to play a role in the modulation of GPCR-activated GIRK channels. In the brain, studies on the RGS6^−/−^ mouse model suggested that RGS6 regulates the deactivation kinetics of GIRK channels in cerebellar granule neurons (CGNs). The impaired gait and locomotor phenotype observed in RGS6^−/−^ mice suggest a more significant role for RGS6 in the cerebellum (Maity et al., 2012). In the heart, experiments on RGS6^−/−^ mice indicated that RGS6 hinders the efficiency and kinetics of muscarinic receptor (M2R)-GIRK coupling and diminishes the amplitude of adenosine receptor (A1R)-GIRK signaling (Anderson et al., 2020).

RGS4, a member of the R4 subfamily, exhibits high expression in the brain and the heart, forming complexes with various GPCRs and accelerating GIRK channel gating kinetics (Chuang and Chuang, 2012). Coexpression of RGS4 in *Xenopus* oocytes decreases the baseline GIRK1/2 current and significantly enhances the proportion of agonist-induced K^+^ conductance (Ulens et al., 2000). Additionally, RGS4 is found to co-localize and directly associate with GABA_B_ receptors immunoprecipitated from the brain (Kim et al., 2014). FRET (Fluorescence Resonance Energy Transfer) experiments suggest a close proximity of GABA_B_ receptors, Gα_o_ subunits, RGS4 proteins, and GIRK2 channels, implying a rapid and specific activation of GIRK2 channels by these modulators (Fowler et al., 2007). In the heart, RGS4-null sinoatrial node (SAN) cells showed a reduction in GIRK channel desensitization and a modification in the kinetics of acetylcholine-sensitive potassium current (Cifelli et al., 2008), indicating RGS4’s role in modulating sinus rhythm.

The overall impact of endogenous RGS proteins on GIRK channel gating suggests that individual RGS knockouts only influence a minor subset of the total RGS influence on GIRK channel gating. The specific contribution of each RGS protein to accelerating GIRK channel gating kinetics appears to be cell-specific, influenced by distinct expression levels of RGS isoforms in the brain and the heart. A thorough investigation of how individual RGS proteins affect GPCR-regulated effectors in specific cell types will enhance the understanding of GIRK channel gating.

### Sorting nexin 27 regulates subunit specification of GIRK channels

In addition to the previously described modulatory mechanisms via changes in GIRK channel gating, total channel activity in neurons is also affected by regulating expression at the plasma membrane. Proteomics studies identified a cytoplasmic protein, sorting nexin 27 (SNX27), associated with a specific subset of GIRK channels (Lunn et al., 2007). SNX27 possesses three operational domains: PDZ (PSD95/Disc large/Zona occludens), PX (Phox Phagocytic oxidase domain), and RA (Ras-association) domains. The PX domain has a specific affinity for PI(3)P and directs SNX27 to the early endosomes (Lunn et al., 2007; Cullen and Korswagen, 2011). The PDZ domain facilitates a direct protein-protein interaction with a Class I PDZ binding motif situated in the C-terminal domain of target proteins (Songyang et al., 1997; Rincon et al., 2007; Lauffer et al., 2010; Cai et al., 2011). In the case of GIRK channels, the PX and PDZ domains play a crucial role in directing GIRK3-containing channels to the early endosomes, resulting in decreased surface expression and smaller GIRK currents (Lunn et al., 2007; Balana et al., 2011).

SNX27b has a putative Ras-association (RA) domain of unknown function, and its interaction through the PDZ domain leads to the reduction of surface expression of GIRK channels (Balana et al., 2011). Deleting the RA domain in SNX27b prevents the downregulation of GIRK2c/GIRK3 channels. Additionally, a point mutation (K305A) in the RA domain disrupts the regulation of GIRK2c/GIRK3 channels and reduces H-RAS binding *in vitro*. The dominant-negative H-RAS (S17N) further hinders the SNX27b-dependent decrease in surface expression of GIRK2c/GIRK3 channels. This finding reveals a novel mechanism involving a functional RA domain and interaction with Ras-like G proteins in modulating SNX27b′s control of GIRK channel surface expression and cellular excitability (Balana et al., 2013).

SNX27 is crucial for maintaining GIRK currents in both the ventral tegmental area (VTA) and substantia nigra pars compacta (SNc) DA neurons (Rifkin et al., 2018). DA neurons in mice without SNX27 displayed diminished GABA_B_R-activated GIRK currents while maintaining normal I_h_ currents and DA D2R-activated GIRK currents. Intriguingly, mice experiencing a substantial reduction in GABA_B_R-activated GIRK currents exclusively in DA neurons exhibited heightened sensitivity to cocaine (Munoz and Slesinger, 2014; Rifkin et al., 2018). These findings suggest that decreased surface trafficking of GIRK channels in VTA dopamine neurons may predispose animals to the effects of psychostimulants (Munoz and Slesinger, 2014). This is supported by the observation that psychostimulant treatment leads to a decrease in GIRK2 plasma membrane localization in these neurons (Arora et al., 2011).

Understanding the intricate details of SNX27 modulation of GIRK channels provides insights into the fine-tuning of cellular excitability of midbrain dopamine (DA) neurons, which plays a pivotal role in the subcellular changes associated with addiction to drugs of abuse. SNX27 has also been associated with various human conditions, such as Alzheimer’s disease, epilepsy, and Down syndrome. Analysis of exome data revealed homozygous mutations in SNX27 among patients exhibiting symptoms of intractable myoclonic epilepsy and developmental delays (Damseh et al., 2015). Down syndrome brains show decreased SNX27 expression and reduced levels of CCAAT/enhancer binding protein β (C/EBPβ), a putative transcription factor for SNX27. Increasing SNX27 levels in the hippocampus of Down syndrome mice improves synaptic and cognitive impairments (Wang et al., 2013). Hence, investigating the role of SNX27 in the brain may offer new therapeutic avenues for diverse neurological disorders.

### Alcohol directly activates GIRK channels through a physical alcohol pocket in GIRK channels

Ethanol exerts diverse effects on both the central nervous system (CNS) and peripheral organs, leading to CNS depression, intoxication, addiction, cardiac arrhythmia, and hepatic and pancreatic dysfunctions (Howard et al., 2014). Initially, ethanol’s actions were thought to result from perturbations in the order of membrane lipids. However, it is worth noting that the effects on membrane lipids are relatively small at clinically relevant concentrations (Peoples et al., 1996). Nevertheless, ethanol at such concentrations can modulate the functions of ligand-gated ion channels, including nicotinic acetylcholine (nACh) (Coverton and Connolly, 1997), GABA_A_ (Wick et al., 1998), glycine (Araya et al., 2021), N-methyl-D-aspartate (NMDA) (Peoples and Weight, 1995), 5-hydroxytryptamine 3 (5-HT3) (Lovinger and White, 1991), ATP receptor channels (Li et al., 1994), as well as voltage-gated Ca^2+^ and K^+^ channels (Treistman et al., 1991). The modulation of these responses by ethanol is contingent upon activating the channels by agonists or membrane depolarization (Treistman et al., 1991; Li et al., 1994).

In 1999, two groups reported the direct activation of GIRK channels by ethanol alone. Ethanol activated both the GIRK1/2, GIRK1/4, and GIRK2 homotetramer channels at physiologically relevant concentrations, independent of interaction with G proteins or secondary messengers (Kobayashi et al., 1999; Lewohl et al., 1999). Other inwardly-rectifying potassium channels are either unaffected by ethanol or inhibited by high ethanol concentrations. Weaver mutant mice, characterized by a missense mutation in the GIRK2 channel, exhibited a loss of ethanol-induced analgesia, suggesting a functional role for GIRK channels in alcohol responses *in vivo* (Kobayashi et al., 1999). Since then, genetic evidence and several behavioral studies have demonstrated the sensitivity of GIRK channels to alcohol and their association with reward circuitry and addiction. GIRK2^−/−^ mice displayed diminished ethanol-induced analgesia (Blednov et al., 2003) and conditioned taste aversion (Hill et al., 2003). GIRK3^−/−^ mice exhibit excessive alcohol drinking and milder withdrawal symptoms (Kozell et al., 2009) and increased ethanol binge-like drinking (Herman et al., 2015) compared to wild-type mice, implying the involvement of GIRK3 in regulating drug effects and the potential susceptibility to addiction (Crabbe et al., 2006). In the human genome, *KCNJ6* was associated with alcohol dependence and hazardous drinking, especially in individuals exposed to early life stress (Clarke et al., 2011). SNPs in noncoding regions of *KCNJ6* were linked to frontal inhibitory control in individuals with alcohol use disorders (Kang et al., 2012) and were found to reduce GIRK2 expression level and increase cellular excitability (Popova et al., 2023). Conversely, upregulated *KCNJ6* in human glutamatergic neurons offers neuroprotection against ethanol-induced hypersensitivity to glutamate, while also promoting elevated intrinsic excitability (Prytkova et al., 2024).

The activation of GIRK channels by ethanol in a G protein-independent manner motivated the effort to characterize the alcohol-binding pocket of GIRK channels. Given the structural similarity between GIRK and Kir2.1 channels (Hansen et al., 2011; Whorton and MacKinnon, 2011), GIRK channels may possess similar alcohol-binding pockets. A high-resolution structure of the cytoplasmic domains of Kir2.1 revealed the presence of a bound alcohol, specifically 2-methyl-2,4-pentanediol (MPD) (Pegan et al., 2006). MPD molecules were found in four analogous solvent-accessible hydrophobic pockets, each pocket comprised of two adjacent tetramer subunits. Notably, this pocket in Kir2.1 has structural features in common with the alcohol-binding protein LUSH in complex with ethanol (Kruse et al., 2003), suggesting that ethanol might bind to the same pocket on inward rectifiers. In both structures, the alcohol pocket is formed by hydrophobic amino acids and polar groups involved in hydrogen bonding.

**FIGURE 4:**
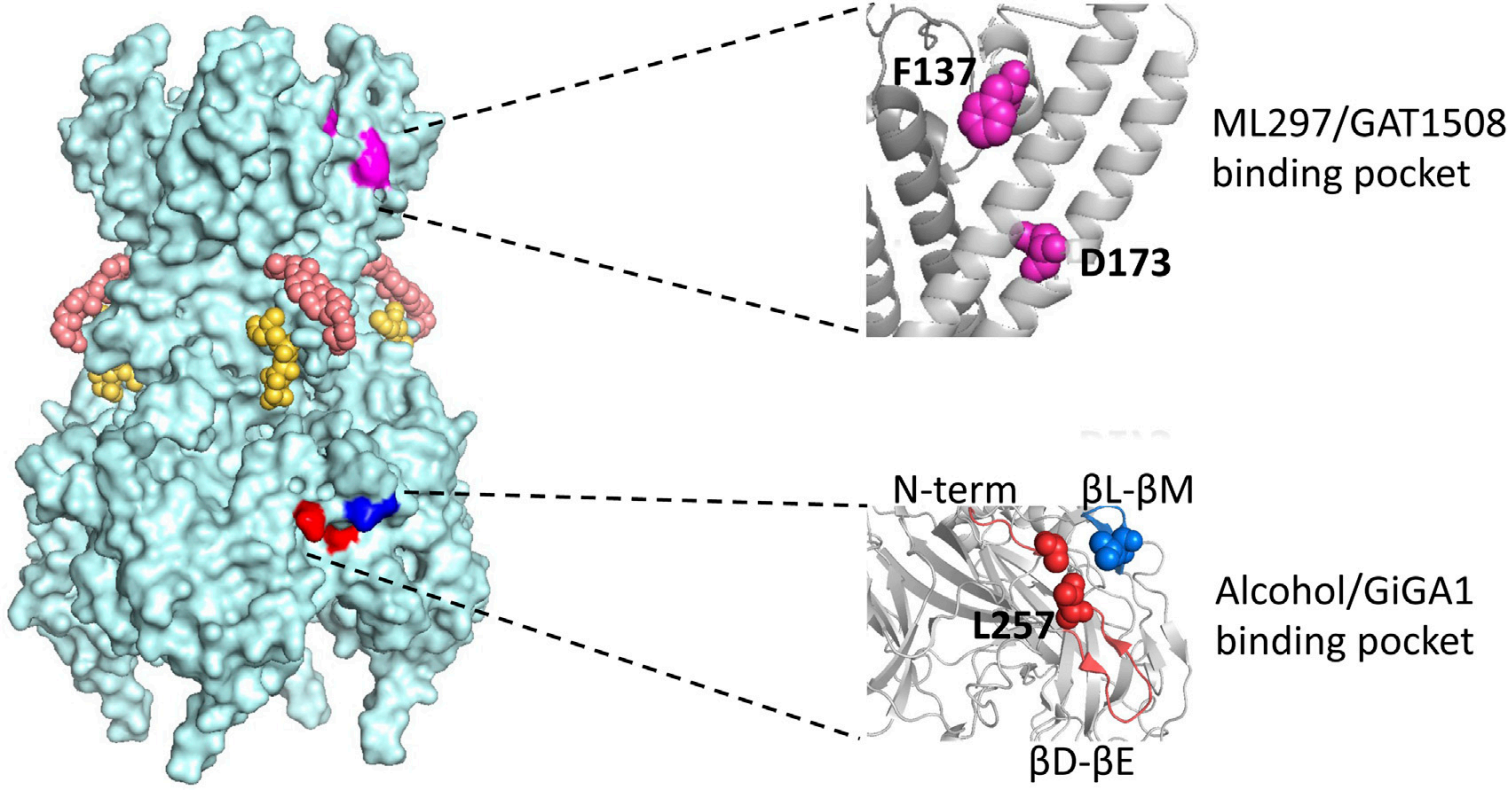
Model of the GIRK2 tetramer with four subunits and locations of various modulatory sites. Corresponding PIP_2_ molecules and cholesterol hemi succinate, CHS, are presented in yellow and salmon, respectively. The region where ML297/GAT1508 potentially bind is highlighted in pink and region where alcohol/GiGA1 bind is highlighted in blue. Expansions of the regions containing key residues implicated in activation by ML297/GAT1508 (top) and alcohol/GiGA1 (bottom) are shown on the right. Amino acids involved in ML297 activation of GIRK1/GIRK2 channels are GIRK1 F137 and D173 (magenta). Key residues forming the alcohol/GiGA1 binding pocket are L257 (red) in the βD-βE loop, I55 (red) in the N-term and L344 (blue) in the βL-βM loop. All residue numbers provided are for mouse GIRK1 and GIRK2 isoforms.

Later, Aryal et al. characterized the equivalent hydrophobic pockets on GIRK2 and GIRK4 channels and identified crucial amino acids for the channels’ interaction with alcohol. Mutations at the conserved L257 of GIRK2, particularly Trp and Tyr, led to a gradual decline in alcohol activation (Figure 4). Similarly, a Trp substitution in GIRK4 at the equivalent position (L252) decreased alcohol-activated currents. The findings suggest that modifying the size of the hydrophobic pocket alters alcohol activation. The proposed model based on mutagenesis and structural analyses suggests that alcohol stabilizes the open conformation of GIRK channels by displacing amino acids in the closed state, particularly at the base of the alcohol pocket (Aryal et al., 2009).

In a study employing an alcohol tagging approach with GIRK2, Bodhinathan and Slesinger (2013) introduced a Cysteine substitution at key residues in a “Cys-less” channel (in which all the intracellular cysteines were removed) and modified with different MTS (methanethiosulfanate) reagents. In this study they showed that modifying a critical Gβγ-binding residue (L344C) with MTS-HE (hydroxy-ethyl; “ethanol”) resulted in a reduction of basal GIRK2 current, while modification of a key residue of the alcohol-binding pocket (L257C) increased the basal GIRK2 current. Additionally, manipulating Gβγ subunit levels consistently affected the rate of MTS-HE-induced inhibition of L344C but had minimal impact on the modification of L257C. These findings suggest that, despite the substantial overlap in the alcohol and Gβγ binding sites, alterations in Gβγ levels do not appear to influence activation by MTS-HE. In summary, these results support a model where the association between Gβγ and GIRK2 L344 in the βL-βM loop (He et al., 2002; Finley et al., 2004), akin to L333 in GIRK1 (Mahajan et al., 2013), precedes alcohol-mediated activation in the pocket (Figure 4).

Like Gβγ-dependent activation, the activation induced by alcohol also relies on the interaction of the channel with PIP_2_. In cells where PIP_2_ levels are depleted using Dr-VSP that removes the 5′phosphate, alcohol fails to activate GIRK channels (Bodhinathan and Slesinger, 2013). Utilizing reconstituted purified GIRK2 channels in liposomes to control membrane components, Glaaser and Slesinger demonstrated that intoxicating concentrations of ethanol (>20 mM) directly activate GIRK2 channels in the presence of PIP_2_ (Glaaser and Slesinger, 2017). Therefore, structural alterations in the PIP_2_ binding interface in GIRK channels represent a crucial gating step for two distinct pathways of activating GIRK channels: a slower Gβγ-dependent activation and a rapid alcohol-dependent activation.

Various studies, including deletion analyses (Lewohl et al., 1999), the identification of an alcohol pocket in X-ray crystallographic structures (Aryal et al., 2009), and alcohol-tagging experiments (Bodhinathan and Slesinger, 2013), collectively suggested that alcohol activation is likely facilitated by the direct interaction of alcohol with the channel. One lingering question is whether the physical alterations in the alcohol pocket are inherently connected to changes in PIP_2_ binding. Lastly, the factors affecting varying alcohol sensitivity among GIRK isoforms remain unknown. Therefore, any therapeutic approach aimed at addressing alcohol use disorder as well as other substance use disorders might want to consider these unresolved questions.

### ML297, GAT1508, and GiGA1–The first GIRK1-containing channel activators

A little more than 10 years ago, no potent, selective GIRK-specific channel activators existed. A high-throughput screening (HTS)-compatible thallium flux assay for G_i/o_-coupled GPCRs using GIRK channels as a readout was developed (Niswender et al., 2008). The HTS involved coexpressing metabotropic glutamate receptor 8 (mGlu8) and GIRK1/2 channels, and the primary screening set yielded around 2000 hits. These hits were tested for their ability to activate GIRK irrespective of GPCR modulation. Further chemical optimization efforts led to the development of ML297 as the first potent GIRK activator (Kaufman et al., 2013).

With an EC_50_ of 160 nM, ML297 demonstrated efficacy comparable to the GPCR activation of GIRK (ref Table 1). It exhibited a preference towards GIRK1/2 channels over GIRK1/4 and is inactive on GIRK2/3 and several other potassium channels (Kaufman et al., 2013). ML297 possesses favorable distribution, metabolism, and pharmacokinetic (DMPK) properties and is centrally penetrant with good CNS exposure in rats (Kaufman et al., 2013). *In vivo*, animals treated with 60 mg/kg of ML297 exhibited an immediate decrease in locomotor and seizure onset, demonstrating significant efficacy in preventing convulsions and a potential therapeutic role in epilepsy (Kaufman et al., 2013) (ref Table 1).

Wydeven et al. identified specific amino acids F137 and D173 in the pore helix and second membrane-spanning domain of GIRK1 as essential for ML297s selective activation of GIRK channels (Figure 4). Behavioral investigations revealed that, beyond its recognized antiseizure efficacy, ML297 reduces anxiety-related behavior without sedative or addictive properties. Conversely, GIRK1^−/−^ mice treated with ML297 did not exhibit anxiolytic effects, suggesting the potential therapeutic applications of GIRK as a target for treating seizure and anxiety disorders (Wydeven et al., 2014) (Table 1).

A few years later, through a combination of chemical screening and electrophysiological assays, Xu et al. (2020) identified a small molecule called GAT1508, a urea-based compound containing a bromothiophene substitution, that activates explicitly brain GIRK1/2 channels rather than cardiac GIRK1/4 channels. Computational methods combined with mutagenesis experiments suggest a crucial role for a conserved TM1 Tyr residue (Y91 in GIRK1 and Y102 in GIRK2), which is established through a network of interactions. Upon binding of GAT1508 to the channel, TM1 Tyr switches from bonding with conserved TM2 Phe residue (F137 in GIRK1 and F148 in GIRK2) to engaging with the TM2 Asp residue (D173 in GIRK1 and D184 in GIRK2). These GAT1508-induced interactions in GIRK channels suggest an allosteric effect on the PIP_2_ binding and channel opening (Xu et al., 2020) (Figure 4).

In brain-slice electrophysiology, GAT1508, even at subthreshold concentrations, directly stimulates GIRK currents in the basolateral amygdala (BLA) and enhances baclofen-induced currents (Table 1). Notably, GAT1508 effectively mitigated conditioned fear in rodents without inducing cardiac or behavioral side effects, suggesting its potential application in pharmacotherapy for post-traumatic stress disorder (PTSD) (Xu et al., 2020) (Table 1).

ML297 and GAT1508 were discovered through high throughput screening followed by mutagenesis and behavioral studies. In 2020, Zhao et al. identified potential modulators of GIRK channels by constructing a structural model of the alcohol pocket in GIRK2 for virtual screening. Utilizing the Kir2.1-methyl-pentane-diol (MPD) crystal structure as a modeling template (Pegan et al., 2006), they virtually screened a library of compounds using the hydrophobic alcohol-binding pocket of GIRK2. Among hit candidates discovered from this structural-based virtual screening and further chemical optimizations, GiGA1 (G protein-independent GIRK activator 1) exhibited high specificity for activating GIRK1/2 channels and demonstrated significantly higher potency than ethanol (EtOH), with an EC_50_ of 31 μM (Zhao et al., 2020), with excellent brain penetration (Table 1). The kinetics of GiGA1-induced currents are comparable to that of alcohol modulation, with rapid activation and deactivation rates.

Mutations in GIRK1 (F137S and D173N) which significantly reduce ML297 activation (Kaufman et al., 2013; Wydeven et al., 2014) did not abolish GiGA1-induced current (Zhao et al., 2020). Computational modeling of the GIRK1/2 alcohol pocket and mutagenesis study reveals critical amino acids for GiGA1 selectivity, R43 in GIRK1, and D346 in GIRK2 (Figure 4). These two residues form electrostatic interactions that stabilize the pocket upon GiGA1 binding (Zhao et al., 2020). Thus, although ML297 and GiGA1 share a common urea backbone, they may interact with different regions of the GIRK channel.

In an *in vivo* epilepsy model, 40 mg/kg and 60 mg/kg of GiGA1 exhibited antiseizure effects (Zhao et al., 2020) (Table 1). In addition, the selective activation of GIRK1/2 channels by GiGA1 holds the potential for addressing other brain disorders, such as alcohol use disorder (AUD) and intractable pain.

In the past decades, advancement in understanding GIRK channel properties has led to the discovery of first-generation drugs that target this ion channel family. Future research should aim to 1) enhance drug specificity by incorporating additional structural-activity relationship (SAR) studies and optimizing existing templates, 2) minimize off-target effects and increase potency, and 3) screen specific compounds for other understudied GIRK channels like GIRK1/3, GIRK1/4, and GIRK2/3 (Zhao et al., 2021). GIRK1/4-specific modulators could be developed for treating atrial fibrillation. Understanding drug mechanisms via biochemical, functional, and structural studies, coupled with improved pharmacokinetics, is crucial for the next-generation drugs. This involves addressing issues like rapid metabolism and targeting challenges for optimal drug delivery to specific organs.

## Summary

Years of extensive research on the activation of G-protein-gated inwardly rectifying potassium (GIRK) channels have elucidated a complex macromolecular signaling framework encompassing both protein modulation (involving G proteins, regulators of G protein signaling [RGS], and sorting nexin 27 [SNX27]) and small molecule modulation (involving membrane lipid phosphatidylinositol 4,5-bisphosphate [PIP_2_], cholesterol, ethanol, and newly identified compounds) (Figure 1). Behavioral investigations involving the four mammalian GIRK subunits within the nervous system, cardiac system, and pancreas have compellingly implicated GIRK channels in a myriad of pathological and neurological conditions.

The elucidation of high-resolution structures and the application of molecular dynamics simulations have substantially contributed to a better understanding of earlier functional observations. These techniques facilitate the visualization of diverse conformational states of GIRK channels. The integration of mutagenesis studies with forthcoming structural insights holds the promise of establishing a more definitive and scientifically robust gating mechanism for GIRK channels.

Further exploration is warranted to unravel the intricate roles of ethanol, SNX27, and RGS in modulating GIRK channels, given their potential multifaceted targets. Advances in expression and purification methodologies are pivotal in enhancing our comprehension of the various GIRK channel combinations. This progress forms a fundamental basis for the development of innovative therapeutic interventions targeting GIRK channels.

A critical Frontier in this domain lies in the identification of small molecule compounds with specificity towards particular GIRK channels. Notably, the discovery of GiGA1 represents a success in the pursuit of structural-based therapeutic interventions targeting GIRK channels.
